# Recent advances in applying machine learning to proton radiotherapy

**DOI:** 10.1088/2057-1976/adeb90

**Published:** 2025-07-23

**Authors:** Vanessa L Wildman, Jacob F Wynne, Shadab Momin, Aparna H Kesarwala, Xiaofeng Yang

**Affiliations:** Department of Radiation Oncology and Winship Cancer Institute, Emory University School of Medicine, Atlanta, GA, United States of America

**Keywords:** proton therapy, machine learning, radiation oncology, medical physics, medical imaging

## Abstract

*Background*. *Objectives*: In radiation oncology, precision and timeliness of both planning and treatment are paramount values of patient care. Machine learning has increasingly been applied to various aspects of photon radiotherapy to reduce manual error and improve the efficiency of clinical decision making; however, applications to proton therapy remain an emerging field in comparison. This systematic review aims to comprehensively cover all current and potential applications of machine learning to the proton therapy clinical workflow, an area that has not been extensively explored in literature. *Methods*: PubMed and Embase were utilized to identify studies pertinent to machine learning in proton therapy between 2019 to 2024. An initial search on PubMed was made with the search strategy ’‘proton therapy’, ‘machine learning’, ‘deep learning’’. A subsequent search on Embase was made with ’(‘proton therapy’) AND (‘machine learning’ OR ‘deep learning’)’. In total, 38 relevant studies have been summarized and incorporated. *Results*: It is observed that U-Net architectures are prevalent in the patient pre-screening process, while convolutional neural networks play an important role in dose and range prediction. Both image quality improvement and transformation between modalities to decrease extraneous radiation are popular targets of various models. To adaptively improve treatments, advanced architectures such as general deep inception or deep cascaded convolution neural networks improve online dose verification and range monitoring. *Conclusions*: With the rising clinical usage of proton therapy, machine learning models have been increasingly proposed to facilitate both treatment and discovery. Significantly improving patient screening, planning, image quality, and dose and range calculation, machine learning is advancing the precision and personalization of proton therapy.

## 1. Introduction

The first proposal of protons as radiation therapy was introduced in 1946 by Robert Wilson [1]. Practical implementation quickly ensued with the first human treatment in 1954 at the Berkeley Radiation Laboratory, and the first therapy center opened in Boston in 1961 [2]. In 1988, the FDA officially approved proton therapy for clinical use. Currently, the National Association for Proton Therapy reports 45 active proton therapy centers located across the United States, with six in development. A recent clinical investigation found that between 2012 and 2021, the total annual number of patients receiving proton therapy increased from 5,377 to 15,829 [3]. The surge in treatment reflects advancements in technology and facilities, along with increased demand due to the unique dose deposition properties of protons [4].

The mechanisms and basic physics of proton interactions have been discussed in detail in previous publications [5–9]. Briefly, as beams travel through tissues, protons undergo inelastic and elastic Coulombic interactions. The primary results of inelastic Coulombic interactions are electron ejection and loss of proton kinetic energy. Proton energy loss dictates range, making an understanding of such Coulombic interactions and their clinical manifestation as the Bragg peak essential. The Bragg peak is the foundational mechanism underpinning the feasibility and efficacy of proton therapy treatment. The shape of the Bragg peak depends on multiple factors including on the fundamental variation of stopping power with energy, the transverse size of the beam, range straggling, beam energy spread, nuclear interactions [10]. In general, the dose deposition by a mono-energetic proton beam is steady with depth until it reaches near the end of its range where the majority of dose is deposited, resulting in a peak known as Bragg peak. Hence, in contrast to a photon beam, protons deposit modest dose initially, with almost no dose deposition beyond the Bragg peak. In this way, a proton beam can be directed precisely such that the Bragg peak occurs within the tumor volume [11].

With precise planning, proton therapy has the potential to spare skin and other organs at risk surrounding tumors. Protons are a preferred treatment option for pediatric patients and those with tumors located near sensitive organs such as the spinal cord, eyes, or brainstem. Lewis *et al* demonstrated that intensity modulated proton therapy (IMPT) better spares the OARs surrounding the tumor and reduces the risk of secondary malignancy in sinonasal cancer patients [12] It is also useful in geometrically complex treatments with challenging dosimetry, such as when treating synchronous bilateral breast cancers. While the proton beams have favorable dosimetric characteristics, each step of the patient care workflow from CT simulation to the completion of the treatment demands great precision and accuracy due to inherent uncertainties including but not limited to tissue density, range uncertainty. As a result, the treatment planning and treatment delivery aspects of proton therapy are less tolerant than photon therapy and can require multiple iterations of each task over the course of a patient’s treatment. Hence, numerous machine learning (ML) applications currently employed in conventional photon radiotherapy and may easily be adapted to proton therapy [13]. At present, such models are used primarily in segmentation, dose calculation, and risk management. Adapting existing ML protocols from traditional photon treatment to proton applications may be expected to improve these processes, enhancing physician efficiency and quality in treatment planning and delivery.

ML, a subfield of artificial intelligence (AI), encompasses models which learn from datasets to generate decisions without being explicitly coded for a repetitive, standard task. ML models may be trained using supervised or unsupervised techniques. Supervised learning provides the model labeled and correctly paired training and test datasets, where the correct output is known for each input [14]. These methods are typically applied to classification tasks where the goal is to map outputs from a given input, such as verifying Bragg peak range or optimizing treatment plans with Knowledge-Based Planning (KBP).

A subtype of ML known as Deep learning (DL) leverages neural networks to interpret data. Inspired by information processing in the brain, neural networks pass raw input data through layers of nodes, each comprised of simple mathematical operations, to create an output [15]. Convolutional neural networks (CNNs) are specialized DL models equipped to process grid-like data. This is beneficial in medical imaging, as CNNs can extract features from the pixels or voxels of patient imaging data across several modalities [16]. Another common form of DL that has been applied in the planning stage of radiotherapy treatment delivery is the Generative Adversarial Network (GAN). This network outperforms traditional DL models when conducting image-to-image translations from unpaired data [17]. The GAN architecture is comprised of two connected neural networks, a generator, and a discriminator. The generator is responsible for synthetic image creation, and the discriminator subsequently evaluates resemblance to the ground truth data. Both networks are simultaneously trained using an adversarial framework, which guides the generator to improve performance with each iteration and produce increasingly realistic data that challenges the discriminator. GANs are particularly useful in complex image translation tasks, such as converting images from one modality to another, such as CT to MRI. U-Nets, CNNs with a U-shaped architecture, are particularly suited for image segmentation tasks because of their strong ability to extract and retain image details across several spatial scales [18]. Such a network can be trained in a few images and performs segmentation-related tasks very quickly.

This review will assess how ML techniques have been implemented throughout various stages of the proton therapy clinical workflow, their drawbacks, and influence on overall proton therapy clinical operations. Despite increased applications of AI in proton therapy, only one study has reviewed the role of AI in proton therapy [19]. While a previous review presents the role of AI in proton therapy according to the tasks (i.e., delineation segmentation, image quality, etc…), the focus of this work is to present the applications of AI in proton therapy in chronological order from patient selection to completion of patient treatment and outcome.

## 2. Methods

**Figure 1.**
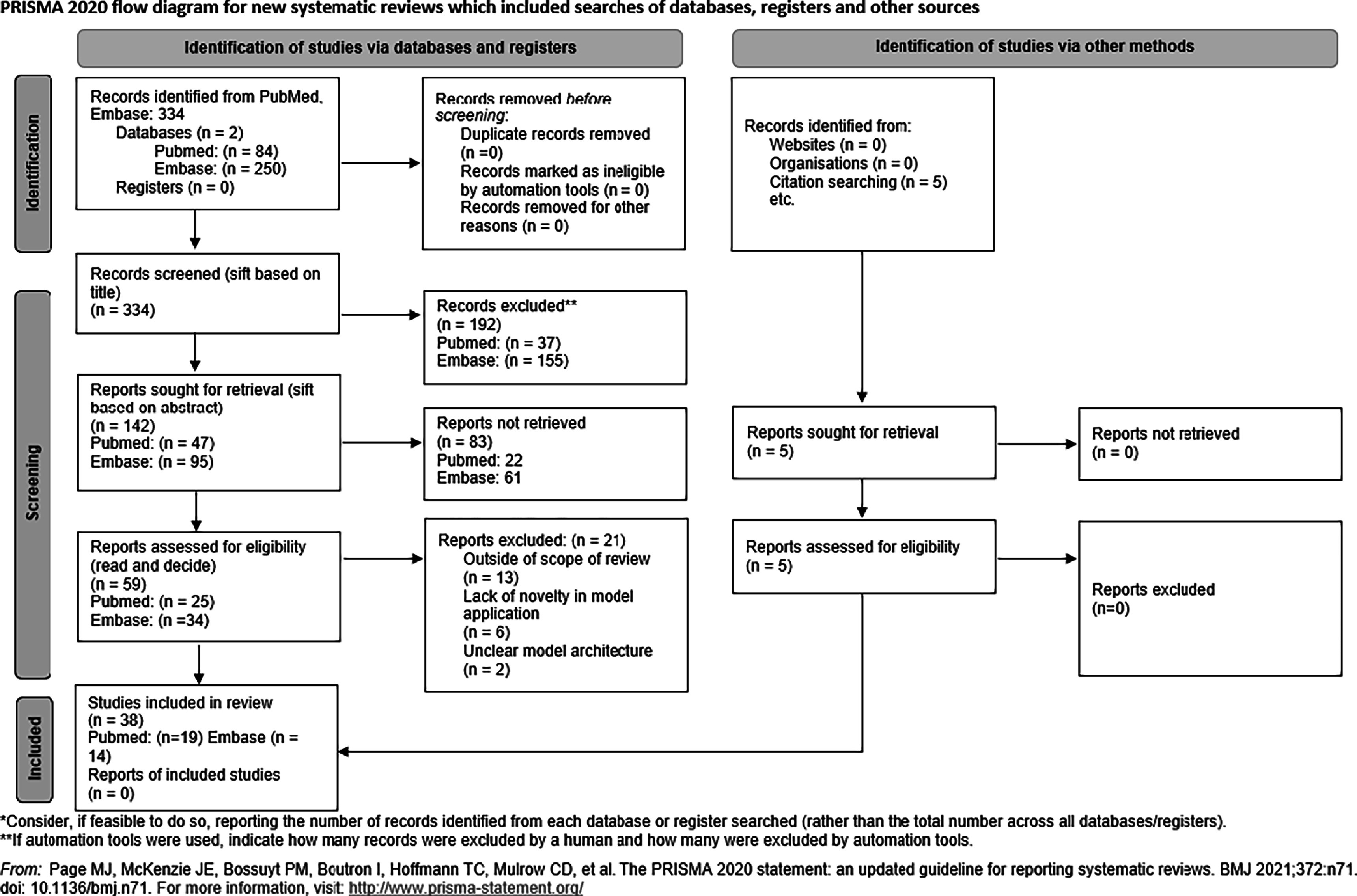
The PRISMA flowchart associated with the systematic review [20]. The number of articles retrieved from each database is outlined respectively for each step of the diagram.

This systematic literature review utilized PubMed and Embase to search for and identify studies pertinent to machine learning in proton therapy. The PRISMA guidelines were utilized to structure the methodological approach (figure 1). The time period of 2019 to 2024 was chosen to capture the most recent signficant advances. Keywords and search strategies were selected to ensure the retrieval of studies specifically addressing the intersection of proton therapy and machine learning, rather than focusing on either topic individually. An initial search on PubMed was made with the search strategy ’‘proton therapy’, ‘machine learning’, ‘deep learning’, with filters including only research articles from 2019 to 2024, returning 84 results. Next (‘proton therapy’) AND (‘machine learning’ OR ‘deep learning’) was searched on Embase, retrieving 546 results. When filtered between 2019 to 2024 and to only research articles, 250 results were retrieved on Embase. Reviews, editorials, technical notes, and articles in any language other than English were excluded from the broad search on both databases. Filtering by title, papers were chosen based on two inclusion factors: explicit application to, or mention of, proton therapy, or a step of the proton therapy workflow, and inclusion of a machine learning model. Assessing by abstract, works irrelevant to chosen components of the proton therapy workflow in scope of the review were excluded. Upon assessing and evaluating full texts for quality, studies were excluded that lacked a clear explanation of model architecture. If multiple studies of the same architecture applied to the same workflow step were identified, only the most recent advancement was included for primary review, while earlier applications were cited for context. An additional 5 studies that met all inclusion criteria were sourced from references of chosen papers. In total, 38 relevant studies have been incorporated into this review.

Despite systematic efforts to ensure an inclusive and robust search, selection bias remains a potential limitation of this review. The search keywords ‘machine learning’, ‘deep learning’, and ‘proton therapy’ were chosen as a catch-all to capture a wide range of pertinent studies at the intersection of these topics. However, terminology specific to certain models, such as ‘convolutional neural networks’, or ‘generative adversarial networks’, was excluded to avoid selectively favoring specific techniques and rather emphasize a broad overview of literature in the field. As a result, some studies that include only these model-specific terms with ‘proton therapy’ in the title may been missed in the search.

## 3. Results

The following section presents a review of articles that highlight the key role of ML in the proton therapy treatment workflow.

– ML for patient selection and predictive outcome modelling in proton therapy (n = 6)
– ML for CT simulation and treatment planning in proton therapy (n = 19)
– ML for *in vivo* monitoring and adaptive treatments in proton therapy (n = 13)

**Figure 2.**
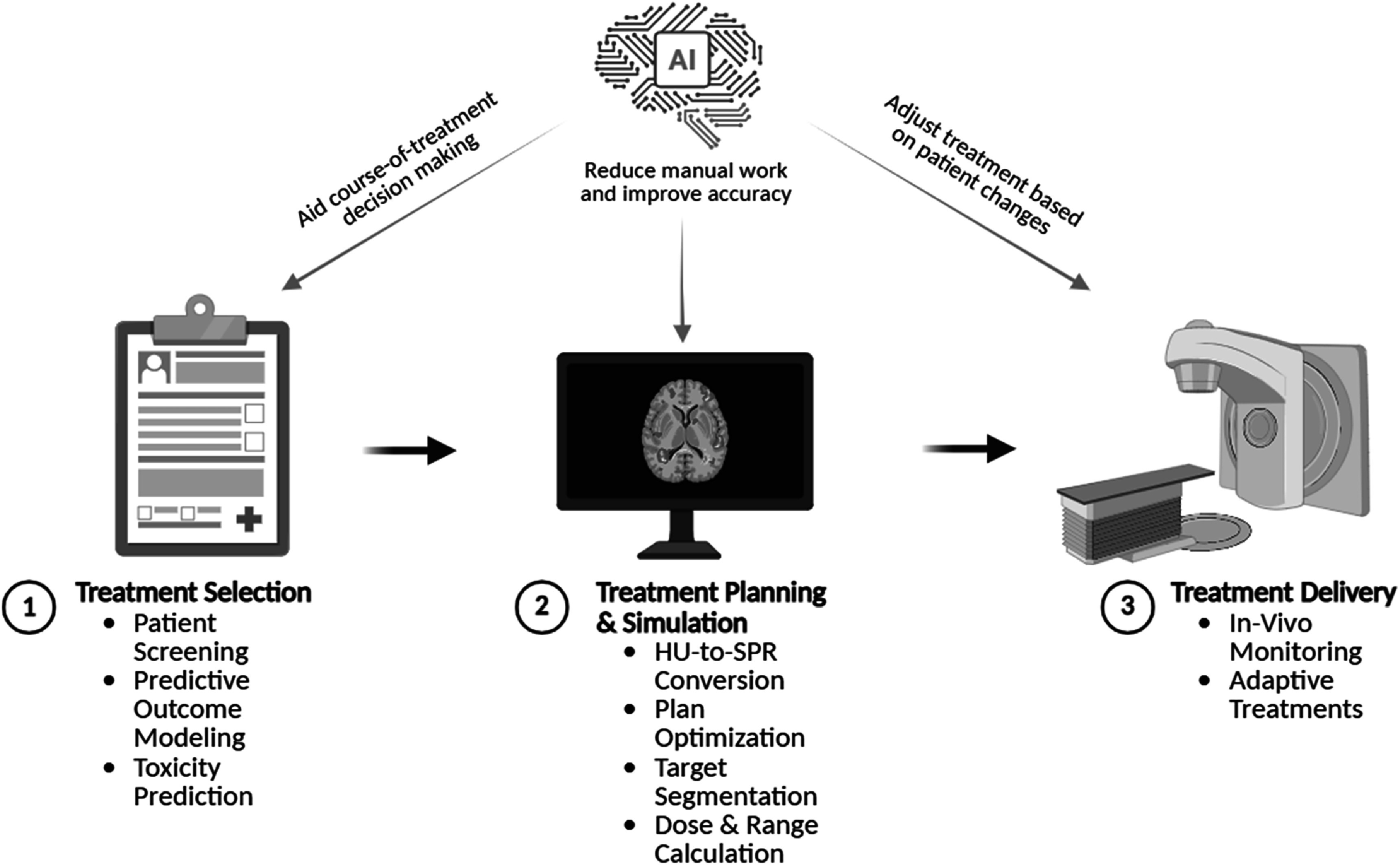
General schematic of the proton radiation therapy workflow with associated tasks [21]. The potential benefits of AI implementation in each step are described.

Figure 2 shows the general workflow of proton therapy treatments along with the potential roles of AI at each step of the workflow. Subsequently, sections 1–3 will provide a brief explanation of the potential area for improvements in each step of the workflow. This will be followed by literature review along with the quantitative results on various AI methods that have been implemented to further enhance the proton therapy workflow.

## 4. ML for patient selection and predictive outcome modeling in proton therapy

ML models are being developed to improve the efficiency of pre-screening processes and determining which modality of radiotherapy will result in the least adverse effects. Pre-selection enables patient-specific data such as tumor characteristics, medical history, and toxicity risk to be compared to an established criteria protocol that assesses whether an individual is eligible for, or will respond better to, proton therapy versus photon radiotherapy. This automated method enables more patients to be pre-screened in a shorter amount of time as well as eliminating any physician bias in the selection process. It is also possible to utilize a predictive model to choose proton or photon therapy based on post-radiation toxicity prediction as a marker of preferability.

### 4.1. Pre-screening for therapy selection

A pre-selection model designed for intensity modulated proton therapy (IMPT) head and neck cancer patients was found to significantly reduce time and labor costs [22]. The model created a fully automated IMPT plan for patients with a prior model-based selection (MBS) approach for photon treatment. A Gaussian naive Bayes classifier for MBS outcome prediction was trained on the dosimetric differences between the IMPT auto-generated plan and the previous photon plan, as well as on the outcomes of the MBS approach. The training process was curated to strongly avoid generating false negatives of IMPT eligible patients. The variables for training compared the differences in xerostomia, dysphagia, and feeding tube dependency for the photon therapy and IMPT plans, and whether or not the MBS approach deemed the individual eligible. After training, a three-dimensional decision boundary was generated based on the xerostomia, dysphagia, and feeding tube dependency measurements. The boundary separated pre-selected patients eligible for proton therapy from those that were not pre-selected by the model and is given by the conditional probability generated from the Gaussian classifier that outlines whether the patient eligible for proton therapy was larger than the decision threshold. Overall results of this work showed that using this pre-selection method can reduce the number of formal selection procedures with involved manual IMPT planning by 67% [22]. Such efficient models provide valuable insight as to whether an individual patient would be selected for proton therapy prior to any other further treatment planning, conserving both time and money.

An alternative model, titled AI-PROTIPP (Artificial Intelligence Predictive Radiation Oncology Treatment Indication to Photons/Protons), features a U-Net architecture and is able to quantitatively assess which treatment method is superior depending on patient factor inputs [23]. This model is able to predict dose distributions for both photon and proton therapy, and use Normal Tissue Complication Probability (NTCP) to determine which therapy would result in the least side effects. This model was employed in clinical cases of patients with oropharyngeal cancer and resulted in 87.4% accuracy of treatment selection based on the parameters given. The average cumulative NTCP for each side effect is not affected by using this model, which only takes around 11 seconds to predict the dose for one modality instead of hours of semi-manual optimization. AI-PROTIPP effectively uses NTCP data to predict dose distributions, select treatment plans, and reduce the time needed for manual comparisons. Similar to AI-PROTIPP, Chen *et al* generated two 3D U-Nets to predict photon and proton doses for patients with localized prostate therapy [24]. They selected NTCP models of grade 2 or higher to determine proton therapy partiality. The deep-learning based dose prediction technique returned with 90%-93.5% selection accuracy based on the NTCP model parameters. These studies demonstrate that DL methods can accurately predict dosage and distinguish NTCP differences between photon and proton plans to select the superior modality for the patient [22–24]. Clinically, ML applied to this area has the potential to decrease the time spent on plan comparison to as little as 5 seconds.

Geng *et al* conducted a multicenter study using a KBP ML model to evaluate and improve radiotherapy plan quality in a clinical trial for non-small-cell lung cancer (NSCLC) [25]. The study aimed to utilize ML to reoptimize 40 IMPT and 40 IMRT (intensity modulated radiation therapy) plans from the RTOG 1308 trial. By comparing the ML-reoptimized plans with the original plans, treatment effectiveness and course of treatment could be compared. Initial model analysis of both sets of the original plans found that PTV coverage varied by as much as 10 to 15 Gy. With KPB model reoptimization, target coverage of the IMPT plans was significantly improved from an original manual PTV of 69.43 ± 0.52 Gy to 70.20 ± 0.29 Gy. This study highlights the KBP model’s utility in QA, and its potential for enhancing radiotherapy plans in multicenter clinical trials. Not only does ML show promise in improving plan quality, but treatment modality decision-making may be assisted through comparative dosimetric analyses of both IMPT and IMRT plans.

A recently developed post-radiation therapy liver toxicity prediction model integrates both photon and proton patient-specific dose distributions [26]. This neural network is able to facilitate the pre-screening process by providing insight on whether photon or proton therapy would be less toxic and more effective in the treatment of hepatocellular carcinoma. An ensemble CNN (CNNE), the model is able to take into account tumor size, liver function, tumor location, and photon versus proton treatment parameters. The predictive model, which was trained on datasets containing the aforementioned variables from patients with hepatocellular carcinoma who received either photon or proton therapy, was able to identify patterns between the variables and post-treatment liver toxicity. The novel CNNE was externally validated using an independent set of hepatocellular carcinoma cases. The proposed model effectively integrated dose-volume histogram (DVH) data with patient-specific liver metrics, outperforming benchmarks with an AUC of 0.78 and an AUPRC of 0.6. Regarding treatment selection, the CNNE identified that patients with baseline impaired liver function are more sensitive to low-dose exposure, suggesting that proton therapy may be safer than photon therapy for these patients. The high precision results demonstrate the potential for ML to assist with synthesizing patient-specific factors and DVH plan data to predict treatment plan efficacy.

### 4.2. Toxicity prediction and strategy

Padannayil *et al* applied a ML model to improve toxicity prediction and to specifically reduce radiation dermatitis for IMPT [26, 27]. Radiation dermatitis, a common side effect of all radiotherapy modalities, is characterized by skin inflammation and redness. It has been reported that up to 67.4% of nasopharyngeal carcinoma patients experience Grade 2 (G2) radiation dermatitis [28]. Therefore, finding ways to mitigate the severity is of high importance. The group developed an unsupervised k-means clustering ML model that was trained on previous head and neck cancer patients treated with IMPT. The mean NTCP values of the training set were 4.97% (G1), 48.12% (G2), and 87.28% (G3). The model created clusters based on dermatitis severity (G1, G2, or G3) and treatment parameters to locate patterns between both factors. By optimizing the distance between clusters, the model was able to group patients according to their likelihood of developing varying grades of radiation dermatitis depending on their treatment parameters. The resultant novel planning strategy integrated with k-means clustering ML was named IMPT-Skin Sparing (IMPT-SS), which reduced mean NTCP from 77% to 43% and lowered skin doses across all dose levels.

Although not currently implementing ML, a noninvasive method to detect early toxicities following proton therapy in pediatric patients has been proposed [29]. Pediatric vertebral irradiation includes the risk of myelosuppression and growth inhibition, enforcing dose minimization. Robust proton therapy planning compensates for range uncertainty, which may lead to excess dose delivered to the vertebral bodies. Ten pediatric patients received 23.4 to 36 Gy of proton body-sparing fractionated craniospinal irradiation (CSI) to the vertebra, and T1/T2-weighted MR scans were obtained before, during, and after therapy sessions. Each MR scan was compared to identify a shift from hematopoietic to decreased metabolically active bone marrow [29]. Such an area of study may be fit for DL or neural networks to be implemented in order to reduce time spent on manual analysis and improve dose deposition predictions.

## 5. Simulation and treatment planning

The clinical workflow of radiotherapy relies heavily on manual inputs and standardized protocols for simulation and treatment planning, critical components that guarantee safe and effective treatment delivery. While current methodologies are effective, they may be time-consuming, require substantial processing power, and fall susceptible to patient-specific variability. With the incorporation of ML, many of these areas may be automated and optimized to tailor each nuance of treatment planning to the individual patient case.

### 5.1. HU-to-SPR conversion (CT/DECT)

The stopping power ratio (SPR) is the ratio of the linear stopping power of the tissue to the stopping power of water [11]. This is an essential parameter in determining how protons will interact with different bodily tissues, such as air, bone, fat, or muscle. The HU from CT images must be converted into SPR values for accurately calculating the proton beam range within a patient’s body. Conventionally, HU-to-SPR conversion is achieved with a calibration curve relating the two units based on known relationships. ML is increasingly being implemented in this clinical step to increase conversion accuracy and reduce systematic errors.

Wang *et al* presented a noise-robust technique to predict relative SPR maps from Dual-energy CT (DECT) images during planning [30]. SPR maps derived from DECT images are frequently degraded due to noise levels and artifacts from physics-based mapping methods. Utilizing ML to reduce the noise when predicting the SPR maps shows great clinical potential. The model utilized a residual attention cycle-consistent generative adversarial network (ccGAN), similar to a CycleGAN, and introduced an inverse SPR-to-DECT mapping to bring DECT-to-SPR mapping close to a 1-to-1 mapping [30]. At simulation, a virtual model of the patient’s anatomy was generated using CT images to optimize the proton plan. Wang *et al* evaluated DECT images from simulations of 20 patients with head and neck cancer with the proposed ML model. A leave-one-out cross-validation strategy evaluated ground truth SPR values which served as learning targets against results from the proposed model. The predicted SPR maps from the model resulted in a mean square error of 2.83% and a MAE less than 3%. Despite the added simulated noise in the DECT datasets, the model still delivered a comparably accurate performance, while the traditional physics-based method faced decreased accuracy due to the noise. Analysis of DVHs revealed that clinical target volumes were below 0.2 Gy with no statistical significance and OARs received around 1 Gy. The results highlight the high accuracy of the predicted SPR maps generated from DECT, despite the presence of noise.

Translating CT numbers into material properties contributes to uncertainty in dose calculations. A physics-constrained DL-based multimodal imaging (PDMI) framework was proposed to bridge the gap between proton range uncertainty and material density of different tissue types [31]. This model integrated physics, DL, MRI, and DECT to generate accurate mass density maps, and built upon previous work by the same group investigating a similar physics-informed DL framework [32]. The prior network successfully generated accurate mass density and relative SPR maps from DECT images from anthropomorphic adult male, female, and child phantoms with unspecified tissue models. Building upon this work, the new study modeled adipose, brain, muscle, liver, skin, spongiosa, and hydroxyapatite bone in phantoms, and MR images were taken of each. From the PDMI framework, an empirical model and various residual networks (ResNet) were generated. Parameters included training with and without a physics constraint, and the utilization of MRI and DECT images vs solely DECT images. Supervised learning further enhanced the ResNet model trained with a physics constraint compared to the ResNest trial without. Additionally, the ResNet utilizing both MRI and DECT images opposed to solely DECT predicted the densities of each tissue most closely to established literature. This novel framework is the first approach to inform DL models with both physics insights and MRI data to derive accurate mass density maps.

SECT, or single energy CT, is less accurate than DECT when used to generate SPR maps. Continuing with similar methodologies to the previous studies [30–32], a residual attention GAN was created to develop DECT images from SECT [33]. The residual blocks forced the model to focus on the disparities between SECT and DECT images. Data from 70 head-and-neck cancer patients with simultaneous SECT/DECT images was retrospectively investigated and used to train the model. A leave-one-out cross validation was used to compare low and high energy synthetic DECT (sDECT) images with ground-truth DECT images. SPR maps were newly derived from the sDECT and compared with those obtained from the retrospective DECT. The sDECT SPR demonstrated an average normalized square deviation (1%) with less noise and artifacts than the ground truth DECT. The clinical target volumes from the DVHs showed less than a 1% difference between the DECT and sDECT doses, highlighting the nearly identical effectiveness of sDECT images in generating SPR maps [33].

A precaution taken when treating pediatric patientsis to avoid extraneous radiation, such as substitution of MR for CT imaging when possible. Harnessing a cycle-consistent generative adversarial network (ccGAN), a group from St. Jude Children’s Research Hospital worked to create synthetic relative proton stopping power (sRPSP) images from MRI sequences, replacing the need for CT images [34]. A dataset comprised of paired CT-converted RPSP maps and MR images from 195 pediatric patients with brain tumors was acquired. 17 combinations of T1-weighted, T2-weighted, and FLAIR MRI were tested, with or without preprocessing, to cover all possible training sequences for the models. The intended strategy of the model transformed patient MRIs into sRPSP images by learning from the paired CT dataset. To assess the performance of the ccGAN and its potential for clinical integration, the team created an online QA tool to guarantee the safe implementation of MR-only proton planning in practice. The generated sRPSP images were converted to sCT, then the QA technique adjusted the sCTs to match a standard reference template created from the training dataset to identify areas where the sCT deviated from the ground-truth CT by >100 HUs. A gamma intensity analysis (10%/3mm) comparing the sCT against the reference CT ‘revealed a strong inverse correlation between gamma intensity and the mean absolute error (MAE) with Pearson coefficients of -0.89 (T1W), -0.93 (T2W), and -0.90 (combined dataset)’ [34]. The group concluded that the ccGAN was able to generate accurate sRPSP images from T1-weighted and T2-weighted MRI images, with the QA tool highlighting regions of inaccuracy to remove unsuitable sRPSP images from clinical use.

### 5.2. Target contouring and segmentation

Manual contouring of OARs and tumors is a laborious, time-intensive task in the clinical radiation oncology workflow. Results have shown that OAR contours created by AI are at least equally consistent withthose completed manually by oncologists with the same level of NTCP [35]. The majority of published ML models related to contouring in treatment planning pertain to conventional radiation methodologies, or do not specify what modality they are addressing. Hence, a very limited number of studies specifically for proton therapy with the aforementioned search keywords were identified. If search criteria were broadened beyond proton therapy as the exclusive modality of choice, extensive literature on ML segmentation would be retrieved. However, it is important to note that the same ML methods dedicated to photon therapy are applicable to proton therapy given that the segmentation tasks are the same, regardless of modality. The performance of ML methods has been extensively studied by reviews for segmentation tasks [36–39].

In the specific context of proton therapy, Nielsen *et al* have evaluated a CNN based on an nnU-Net to contour OARs in a Danish pilot trial [35]. While the median DSC for manual contours completed by physicians was 0.68, those completed by the CNN achieved an improved DSC of 0.85 [35]. A highly relevant study on general radiotherapy explored how DL models can be implemented in the identification and delineation of OARs in head and neck cancer treatment [40]. A region commonly treated with proton therapy, precise contouring of head and neck cases is crucial to ensure that radiation is sufficiently covering and concentrated on the tumor. Van der Veen *et al* assessed a CNN to automatically delineate 16 head and neck OARs from patient CT or MRI scans used in planning [40]. While manual contouring of all OARs took between 22 and 44 min, the CNN was able to complete the same task in 3 min. To review and correct the 16 OAR contours, it took the CNN an average of 23 min while manual completion of the same task spanned 13–33 min. Incorporating the time for initial delineation, implementation of the CNN saved 6–19 min per patient compared to conventional manual delineation.

### 5.3. Synthetic image generation, auto-planning and treatment plan optimization

Synthetic data are artificially generated data that are comparable to real-world data, which, in turn, can be used to increase the size of the datasets to improve the performance of the ML models. Synthetic CT (sCT) scans can be generated from the cone-beam CT (CBCT) scans to enhance dose calculation and treatment planning in adaptive settings.

A 3-dimensional cycle generative adversarial network (CycleGAN) was trained on CT and MRI data pairs from a training cohort composed of patients with base-of-skull tumors [41]. The clinical study consisted of 50 patients, split into two non-overlapping cohorts, one for training and one for study purposes. Post-training, synthetic CT (sCT) scans were generated by the model and compared against the initial CT scans where the mean error ranged from 38.65 Hounsfield units (HU) to 65.12 HU. Proton plans with 2 beams each were generated based on the initial CT scans as well as the sCT images and were compared based on DVH endpoints and proton distal range along the beams. Quantitative results showed that the model-generated sCT was in agreement with the ground truth CT; the dosimetric evaluation of the DVH endpoints did not have statistically significant differences, and 96% of the proton ranges were clinically accepted.

A previous study showcased a CycleGAN capable of generating clinically-acceptable sCT images and highlighted the potential for MRI-based proton treatment planning [41]. Chen *et al* subsequently utilized a similar approach on a larger dataset of 206 nasopharyngeal carcinoma patients, with two conditional GANs trained on T1-weighted MR-CT image pairs [42]. The images were paired through deformable image registration. The two model architectures consisted of a 3D U-Net augmented with residual connections and an attentional generator, as well as a 2D-GAN with a 2D U-Net as the generator. The minimum gamma passing rate for all generated sCT images was above 97%, and MAEs between the sCT and ground truth for both the 2D and 3D GAN models were ~64 HU. The sCT images created by the GANs were of clinical dosimetric accuracy, indicating that advanced deep CNNs may further improve MR-based sCT image generation in proton therapy planning [42].

Deep-learning generated sCT from paired MRI for IMPT has been subsequently built upon by Zimmerman *et al* [43]. A DL model capable of generating sCT images independent of MRI sequence data was created in 2022 [43]. 47 meningioma patients treated with pencil beam scanning (PBS) therapy were divided across training, validation and test groups. MRI sequences combined with planning CT (pCT) data were used to train a 3D U-Net framework with ResNet-Blocks, a type of CNN block in which the output from one layer is added into a deeper layer. The training outcome was assessed by metric, dosimetric, and spot difference map accuracy in comparison to the original treatment plans. The synthetic dose parameters of the proposed model agreed within 1% of the real-world original plans, and 98% of the spots on the spot difference maps had less than 1 cm difference from the original plan. The innovative MRI-independent sCT generator created by Zimmerman *et al* suggest that ‘the training phase of neural networks can be disengaged from specific MRI acquisition protocols’ [43].

KBP utilizes data at the forefront of treatment planning, incorporating clinicians’ knowledge into precise models. Post-processing and dose mimicking are critical steps to create clinically acceptable ML-planned IMPT plans. Borderias-Villarroel *et al* [44] investigated the quality of such plans utilizing four different KBP pipelines. The four models were generated from two sets of conditions: post-processing (PP) or no post-processing (NPP) and isodose-based (IBM) or commercial RayStation dose mimicking (RSM). The study trained 11 dose prediction models using data from 60 patients with oropharyngeal cancer, achieving similar target coverage (D98% ≥ 95%) for both high-dose and low-dose CTVs. Additionally, all four pipelines improved OAR dosage compared to manual plans. Both pipelines with post-processing (PP-RSM and PP-IBM) reduced median dosage to the oral cavity by over 6 Gy, to the parotides by 5 Gy to the parotids, and to the pharyngeal constrictor muscles by 4.5–5 Gy. On the other hand, post-processing after the initial ML output appeared to compromise the robustness of the plan. This was particularly evident in high-dose CTVs, where post-processing created steep dose gradients which resulted in cold spots and underdosage in 65% of the plans. Overall, the KBP pipeline combination of RayStation dose mimicking and no post-processing (NPP-RSM) produced the IMPT plan of the highest robustness with sufficient OAR sparing.

A group from the Netherlands employed a machine learning optimization (MLO) algorithm to generate IMPT plans for oropharyngeal carcinoma patients [45]. Compared to manually optimized IMPT plans, the MLO-generated plans achieved adequate robust target coverage in 23 of 25 patient plans. MLO plans showed significantly increased average OAR dosages in the pharynx constrictor muscles and cervical esophagus, which requires further improvement Utilizing ML to fully auto-plan proton therapy while taking into account all quality assurance (QA) measures is difficult. As a QA measure, incorporating realistic spot delivery error predictions into treatment planning ensures adequate robustness of the plan. While attempts to implement ML into this step have been made, current studies have shown a decrease in robustness of the model-predicted plans [46].

### 5.4. Dose prediction, deposition & verification

PBS dose calculation may be inaccurate due to approximations used to work around heterogeneities in tissue. Doses are computed based on the water-equivalent path length along the proton beam’s center, and the medium is treated as infinite and homogeneous. However, in clinical reality tissue is rarely homogenous, leading to range uncertainties and misrepresentations of multiple Coulomb scattering. Despite being the current gold standard and extremely accurate, Monte Carlo (MC) dose calculation suffers from slow processing due to tracking each individual particle. To mitigate the tradeoff between accuracy and time when utilizing PBS dose calculation or MC simulations, incorporating ML models may improve the shortcomings of either method.

To boost the accuracy of PBS dose planning while maintaining a shorter time scale, Wu *et al* created a DL model that converts a PBS dose to a MC dose using the initial PBS data and the CT images [47]. The model was trained on data from four tumor sites: head and neck, liver, prostate, and lung. The average gamma passing rate, a QA measure, was >88% between the converted PBS dose and the MC dose, and the conversion time was less than 4 seconds. Such results demonstrate high accuracy and rapid speed, combining the strengths of both planning techniques. Training the model on data from all tumor sites together and using the dose distribution of each individual beam as input yielded the best performance for all four tumor sites [47].

Proton Source Model Commissioning (PSMC) is a critical component of the planning workflow which aims to optimize the match between calculated dose and delivered dose in PBS therapy. Presently, PSMC ensures accurate dose calculation via MC simulations. Nominal energy refers to the average or expected energy value of the protons in the simulation, while the energy spread is the range of energies around the nominal energy. Calibrating the nominal energy and energy spread parameters in the PSMC is difficult as these parameters are not able to be easily solved from an equation. To facilitate these calculations, a CNN known as ‘PSMC-Net’ was developed [48]. PSMC-Net was trained on a range of 33 clinical-level energies, from 75–225 MeV. For each of the 33 energies, a dataset was generated with 15 nominal energies, 10 spreads, and the corresponding 150 calculated depth doses. 130 of the data pairs were used for training, 10 for validation, and 10 for testing. Results showed that the gamma pass rate between the MC and measured depth doses was 99% when PSMC-Net was implemented. Without PSMC-Net, the gamma pass rate was ~54%. This difference in plan quality showcases the improvement in efficiency of the PSMC process when incorporating a CNN-based proton source model.

Calculating the output factor (OF) is an additional step in beam commissioning and dose calculation. The OF is a patient-specific correction factor which adjusts the dose delivered based on the characteristics of the treatment field. Monitor units (MUs) are is a measure that quantifies the amount of radiation delivered by the machine. MUs are a function of the OF, as the OF is necessary to modify the basic dose rate of the machine. Both of these components are necessary in the beam commissioning planning step to ensure the prescribed dose of radiation is delivered to the patient. A previous study by Sun *et al* utilized ML to derive MUs as a secondary QA tool for OF measurements in 2018 [49]. The group used three supervised learning models; Random Forest, XGBoost, and Cubist. Grewal *et al* more recently developed a DL technique to predict OF and MU based on measured patient QA data [50]. The group implemented MATLAB-based supervised DL algorithms (Gaussian process regression and shallow neural networks) to calculate the OF based on the standard empirical model [50, 51]. The models were trained on a randomized patient QA dataset consisting of range, modulation, field size, and measured OF data. 90% of the data was used for training, while the remaining 10% was allocated for testing. The trained model successfully predicted the OF from three input parameters: range, modulation, and field size. The accuracy of both the Gaussian regression and shallow neural network were higher than the empirical model for each parameter (± 2% and ± 3% difference) [50].

Linear energy transfer (LET) describes the amount of energy, or radiation, transferred per unit length (keV/μm) as an ionized particle travels through a medium. MC simulations are utilized as the gold standard to predict yield and LET at a certain depth of the proton treatment plan to assess biological effectiveness. These simulations are difficult to validate experimentally. Stasica *et al* utilized the power of Timepix, a high spatial, high contrast resolving pixel read-out chip working in single photon counting mode which was assessed in water [52]. The introduction of such a measurement method enhanced by AI provides experimental data to validate LET spectra in proton therapy. The neural network model achieved over 95% accuracy in proton recognition, in which the LET spectra experimentally measured were mostly consistent with the results of MC simulation. The detector was able to capture depositions from a fraction of keV/μm to about 10 keV/μm in the mixed radiation field. The simplicity of this technique has promise of clinical implementation in cases where minimizing OAR exposure is critical and stands as one of the first LET studies implementing ML in proton applications.

Gao *et al* employed a similar method to verify LET via an entirely synthetic computational approach for proton stereotactic body radiation therapy (SBRT) [53]. To date, this is the first time a KBP methodology was adopted in the context of LET distributions. Utilizing the dose distribution map, the DL framework predicted the LET distribution map of the protons to better estimate the relative biological effectiveness (RBE). The framework used data from 50 prostate cancer patients receiving proton SBRT, and featured a 5-fold cross-validation method, dividing the patient dataset into five subsets. In each iteration, four subsets were allocated for training and the remaining one was designated for testing, ensuring each patient’s data was tested exactly once. Gao *et al*’s DL model is comprised of two sub-networks; two U-net-based generators that generate a synthetic LET map image derived from the dose map image, and two discriminations which minimize judgement error [53]. The supervised CycleGAN model results surpassed those of other GAN-based models with a mean absolute error (MAE) of 0.096 ± 0.019 keV/μm. This model may significantly improve proton therapy plan quality and efficacy through RBE optimization, supported by the availability of accurate LET data.

Recently, similar results were shown with a DL-based model which calculated dose-averaged LET using CT images and dose-to-water data [54]. This resulted in real-time biological dose evaluation and LET optimization. 275 proton SBRT plans were analyzed, rendering 1100 fields. These were split into various training, validating, and testing subgroups, and a 3D Cascaded UNet model was created to generate patient-specific LET distributions from patient anatomy data extracted from CT images and the dose-to-water data. The accuracy of the generated LET spectra was held against the gold standard MC result through voxel-based MAE and gamma analysis. The model took around 100 ms on a Nvidia A100 GPU to generate a LET calculation, which yielded an MAE of 0.94 ± 0.14 MeV/cm and a gamma pass rate of 97.4% [54]. The results from this study demonstrate the potential for DL models to accurately calculate LET distributions more quickly than current manual and MC methodologies under the tested conditions. However, as with all studies, it is of importance to note that findings are based on a specific dataset and experimental setup. Therefore, further validation in clinical scenarios involving complex heterogeneities is required to establish generalizability. Nonetheless, this is one of the first models to fully generate an LET distribution and has the potential to be applied clinically with further testing [54].

### 5.5. Range prediction, calculation & verification

Utilizing ML to hybridize dosimetric and range predictions, artificial neural networks (ANN) have been developed with the ability to predict six variables related to dosimetric parameters: proton beam spot size in the *x*-axis, *y*-axis, major axis, and minor axis, and relative x and *y*-axis positional errors [55]. All of the ANN models utilized a multi-layer perception (MLP) network with one input layer, three hidden layers, and an output layer. Trained on data from 9000 proton spots, all predicted spot size location and positional errors were evaluated against scintillator-measured data as reference. Scintillators are materials that absorb ionizing radiation, or the energy from charged particles, and convert it into short bursts of visible photons or UV light. The ANN models resulted in lower prediction errors compared to the scintillators and demonstrated excellent beam spot size predictions as well as positional errors.

ML has also been used to generate synthetic CT (sCT) images from cone-beam computed tomography (CBCT) of the abdominal cavity to estimate proton ranges [56]. Bowel gas pockets were intentionally included to support clinical realism and address a common imaging challenge. A robust dataset composed of the CBCT, same-day CT, and pCT of 81 pediatric patients was used for training, testing, and validation. The proposed hybrid model combineed a CycleGAN with deformable image registration, leveraging superior image synthesis with strong pCT-to-CBCT registration. The CycleGAN portion generated the geometry-weighted components of the sCT, and the deformable image registration portion generated the intensity-weighted components. The benefit of this hybrid approach is improved sCT quality by implementing two different models with strengths tailored to each component. The hybrid sCT presented improved gamma pass rates (99.7±0.8% vs 98.4±3.1%/98.3±1.8%), indicating a dosimetric advantage. Additionally, the calculated proton beam ranges which crossed gas pockets demonstrated increased accuracy (90^th^ percentile error in 80% distal fall-off, 1.8±0.6 mm). This model shows promise in CBCT-based proton range verification in pediatric patients receiving abdominal cavity radiation.

An alternative method to estimate proton beam range is the use of optical camera systems equipped with scintillators. Scintillators are excellent candidates for dosimetric or range QA measures in radiation therapy. As passive detectors, scintillators provide a non-invasive, safe option for clinical usage. Additionally, the devices are able to provide real-time feedback with high spatial resolution provided by the sensitive ability to capture minute light emissions. They are cost-effective, adaptable to different radiation energies, and generally compatible with optical imaging systems. Lee *et al*’s combined scintillators with DL as a QA measure for both range and dose [57]. The group remarks on the difficulty previous methods have had with deriving dose distribution analytically from scintillation images manually due to quenching and optical effects. Their study introduced a 2D residual U-net DL approach for beam range and spread-out Bragg peak (SOBP) predication using a scintillation light distribution (LD) captured in a water phantom. These predictions were reached via 2D map conversion of the LD maps into dose maps. The training and validation set was comprised of 8659 image pairs generated via MC simulations with varying proton beam conditions [57]. The model showed high accuracy with beam range and SOBP width resolutions of 0.02 mm and 0.19 mm, respectively, and deviations of less than 0.1 mm and 0.8 mm from reference simulations. Results demonstrated good agreement in gamma analysis, validating the feasibility of this DL-based QA method in clinical practice.

## 6. In-vivo monitoring and adaptivity in proton therapy

Continual adaptation and replanning are crucial components of both photon and proton therapy, and demonstrate reductions in NTCP and improved target coverage [58]. The two primary adaptive therapy techniques include offline replanning, which occurs when a new plan is generated between fractions, and online replanning, which occurs while the patient is on the treatment table. ML has the potential to benefit both areas of adaptive proton replanning, which are time- and resource-consuming.

### 6.1. Anatomy changes

As noted by Wang *et al,* the sculpting ability benefits of IMPT are countered by increased sensitivity to anatomical variations during and between treatment sessions [59]. Such variations may result in suboptimal delivery to the tumor or increased dosage to OARs. Adaptive anatomy planning is resource-intensive and usually requires additional CT simulation. DL may improve this area of clinical workflow by allowing sCTs to be derived from offline on-treatment MRI images to calculate dose delivery based on daily anatomy, as well as flagging cases which may require adaptation.

The group developed a novel CycleGAN model with self-attenuation with abilities to identify pediatric patients that would benefit from adaptive replanning [59]. The goal of creating such a model, opposed to utilizing a conventional CycleGAN, is to assess if the addition of self-attenuation generates more accurate sCTs for children with brain tumors between the ages of 2 and 21. The goal of introducing self-attenuation to a conventional CycleGAN is to enhance boundary delineation of bone-air tissue interfaces, specific to common pediatric brain tumors, and to reduce noise. The training dataset was composed of both CT and T1-weighted MR images of 125 children with brain tumors. Seven patients between the ages of 2 and 14 that underwent adaptive planning due to anatomy changes discovered via MRI during proton therapy comprised the test dataset. Results were obtained by comparing the MRI taken during proton therapy with the model-generated sCT and the replanning CT (ground truth). The HU MAE with the self-attenuation CycleGAN was 65.3 ± 13.9 versus 88.9 ± 19.3 for the conventional CycleGAN, demonstrating improved accuracy by the proposed model. The self-attenuated model also demonstrated improved gamma passing rates and appropriately triggered plan adaptation in all test patients.

Similar to the study utilizing a CNN to contour OARs in conventional radiation planning [40], Elmahdy *et al* utilized a CNN to automatically segment the bladder from CT scans of IMPT prostate cancer patients online [60]. This study focused on online adaptive IMPT, where the patient’s anatomy is continuously tracked to generate real-time treatment adjustments in the case of observed changes. In the case of prostate cancer, the goal of the model was to monitor changes in bladder or prostate shape or location to minimize OAR dosage. Particular attention was paid to accurate segmentation of the bladder and adjacent structures (prostate, seminal vesicles, and lymph nodes). The Dice Similarity Coefficients of the bladder segmentation network on two separate datasets were 88% and 82%. Simultaneously, MSD of the prostate, seminal vesicles, and lymph nodes were calculated as 1.29 ± 0.39, 1.48 ± 1.16, and 1.49 ± 0.44 mm respectively. Such a novel registration pipeline is therefore a potential application to both planning and adaptive segmentation of OARs for IMPT.

The proposed CNN by Thummerer *et al* is able to predict deformation vector fields which are required for accurate contour propagation by detecting spatial transformations between the reference CT image and the real-time treatment imaging [60]. The CNN was trained on manually delineated images and corresponding deformation vector fields of 20 prostate cancer patients. As the network makes the continuous comparison between the two images, this enables adaptations in the treatment plan to be generated based on the patient’s anatomical variations during the treatment session. Their results found that the combined CNN and image registration technique improved the target delineation accuracy significantly with a dice similarity coefficient of 88% compared to manual attempts. The CNN-propagated contours met dose coverage constraints in 86%, 91%, and 99% of cases for the prostate, seminal vesicles, and lymph nodes, respectively, and that 80% of the automatically generated treatment plans were directly usable without manual correction [60]. Such findings may improve clinical efficiency and potentially reduce adverse side effects by sparing healthy tissue.

CBCT is used to verify patient positioning prior to radiation therapy and are valuable for adaptive planning as they provide daily insight regarding patient anatomy. 4D-sCTs may be generated from 4D-CBCTs, which are similar to traditional 3D modalities but feature a time-resolved aspect. The combination of the unique nature of the Bragg peak and the clinical importance of accounting for thoracic respiratory motion during treatment motivates the clinical usage of 4D-CBCTs in adaptive proton planning. Despite the clear benefits of utilizing 4D-CBCTs, they are frequently afflicted by imaging artifacts not seen with diagnostic CTs that prevent accurate dose calculations. Such an area is fit for DL to adjust inaccuracies that will allow 4D-CBCT-based adaptive proton dose calculations.

A group based in the Netherlands implemented a U-net-like deep convolutional neural network (DCNN) to convert sparse view 4D-CBCTs into 4D-sCTs [61]. The DCNN architecture was previously established by Spadea *et al* [62]. The novel DCNN was trained on 4D-CBCT data from 45 thoracic cancer patients to generate 4D-sCTs. With the MA-ROOSTER reconstruction algorithm, unprocessed CBCT projections collected during a single treatment fraction were utilized to reconstruct the 4D-CBCTs retrospectively for each patient. Employing the model resulted in 4D-sCTs with ‘average MAEs of 48.1 HU (single phase) and 37.7 HU (average)’, and ‘gamma pass ratios of 92.3% (single phase) and 94.4% (average)’ [61]. There was a high agreement between ground truth 4D-CT and 4D-sCT clinical target volume, but ‘larger dose differences were observed in mean doses of OARs’ [61]. The group reported that ‘the dosimetric results show a comparable performance of 4D-sCTs with respect to 3D-sCTs, suggesting the potential suitability of sparse view 4D-CBCT-based sCTs for proton dose calculations in adaptive proton therapy workflows’ [61].

SWFT-Net is a DL framework that focused on adjusting spot weights and optimizing treatment plans online [63]. The primarily goal of the network is to speed up treatment plan adjustment as anatomical changes are detected in patients with daily imaging. The model is able to adjust proton therapy treatment plans under a second with the NVIDIA GeForce RTX 3090 GPU, and was tested extensively on 1,706 head and neck cancer samples. When adjusting beam settings adaptively, the normalized root mean square errors (NRMSE) for three different increasing spot weights were 0.41%, 1.05%, and 2.04%. The mean gamma passing rate was above 95% for all three spot weight datasets of interest. Such a model is a step towards a fully online adaptive proton therapy workflow.

### 6.2. Range verification

Protoacoustic signals in proton therapy are acoustic waves generated when protons interact with tissues. According to the Bragg peak, protons suddenly and rapidly deposit energy at a certain depth, causing a local transient increase in temperature and pressure [64]. This minute expansion generates protoacoustic signals, which may be detected with ultrasound sensors. The clinical relevance relates to recreation of the Bragg peak and real-time monitoring of proton beam range, ensuring that the beam is depositing at the intended target depth. Protoacoustic signal denoising is quickly gaining traction in the field of proton therapy research, and ML techniques are readily being incorporated.

While protoacoustics are capable of detecting the Bragg peak location *in vivo*, the technique requires a large dose delivery to acquire an adequate signal-to-noise ratio (SNR) and large number of signal averages (NSA). Such large doses are not suitable for clinical use. A novel DL model demonstrated enhancement of the SNR of protoacoustic measures as well as improving Bragg peak location identification accuracy in range verification scenarios [65]. Three accelerometers, two of low accuracy and one high, were placed on cylindrical polyethylene phantoms to record protoacoustic signals during simulated treatment. On each accelerometer, 512 raw signals were obtained. Device-specific stack autoencoder (SAE) denoising models were trained to denoise noisy input signals (averaging 1, 2, 4, 8, 16, or 24 raw signals). Clean inputs with high NSA were generated by averaging 192 raw signals. Two models were tested: one supervised during training and the other unsupervised. When assessing Bragg peak range uncertainty after testing, the supervised SAE outperformed the unsupervised SAE at low NSA levels, decreasing MSE by 80%–85% and increasing SNR by 59%–87% at the lowest NSA of 1. Clinically, SAEs show promise to denoise protoacoustic signals better than conventional Gaussian low-pass filters.

Expanding upon the range of applications of ML in online range monitoring, Jiang *et al* proposed a novel architecture capable of denoising acoustic, protoacoustic, and electroacoustic signals both quantitatively and qualitatively [66]. Radiation-induced acoustic imaging generally requires a substantial number of recorded frames to achieve a satisfactory dose deposition average, exposing the patient to increased radiation. The proposed model is a general deep inception convolution neural network, or a GDI-CNN. The model features radiation-induced acoustic signal denoising capabilities, which results in a reduced number of frames required for averaging. The inputs of the model were the few-averaged noisy signals from a protoacoustic radiation therapy trial, which in turn outputs high-SNR denoised signals. After training and testing, GDI-CNN performance was assessed in comparison with 1500-frame-averaged experimental protoacoustic signal results. The model achieved proton range accuracy with significantly lower MSE (0.0011 ± 0.0003) and higher PSNR (29.604 ± 1.2060) than benchmark models: U-Net (MSE: 0.0036 ± 0.0014, PSNR: 24.809 ± 2.0970) and U-Net-Lite (MSE: 0.0024 ± 0.0014, PSNR: 27.072 ± 2.900). Additionally, GDI-CNN demonstrated improved signal quality for real-time proton therapy monitoring, with higher SSIM (0.9905 ± 0.0014) compared to U-Net (0.9843 ± 0.0031) and U-Net-Lite (0.9821 ± 0.0034), which indicates at least comparable reconstructions.

Compton imaging, originally utilized in astronomy to locate planetary radiation sources, has been adapted for medical applications. In proton therapy, prompt gamma imaging (PGI) serves as an alternative method to verify range and classify treatment deviations. Similar to protoacoustic signaling, target nuclei emit prompt gamma-rays as they de-excite after proton beam irradiation. These detectable secondary particles captured by Compton cameras provide near-real time measurement of proton penetration depth. Lerendegui-Marco *et al* explored the i-TED detector (an array of Compton cameras) for range verification in proton therapy [67]. When combined with ML to improve the SNR, the detector is capable of discerning high-energy gamma rays emitted in tissue. Previous literature has shown that the distribution of secondary gamma rays closely reflects the proton dose distribution at the tumor site, making it a beneficial target for range verification [68]. The group successfully implemented a novel event classification algorithm to filter out high-energy gamma ray events without complete energy deposition. The model uses TensorFlow with four fully connected layers, applying rectified linear activation functions for feature extraction and a sigmoid activation function for classification. Such model is the first to improve signal-to-background ratio by as much as twofold. Concluded from MC simulations and PMMA phantoms, the ML-aided i-TED detector reduced the F80% deviation to 1.3 mm, indicating improved precision of dose delivery. A spatial resolution of 4.4mm in a 4-MeV window at 511 keV was achieved, and the model successfully halved the number of neutron-induced signals. In the energy range of 0.4 to 2.5 MeV for 511 keV gamma rays, i-TED demonstrated an efficiency of about 85%, up to 1.6 × 10^−3^ per emitted gamma-ray, surpassing the existing Compton camera. Further studies are intended to be conducted at the cyclotron at CAN (Sevilla) and with clinical beams, demonstrating the possibility for ML models to improve multiple facets of range verification.

While the previous study analyzed the application of ML to physical hardware, Pietsch *et al* investigated the application of CNNs to MC simulations to automatically classify and detect treatment deviations [67, 69]. Manual interpretation of spot-wise range shift data is laborious and not feasible in repetitive routine application. To address these concerns, a CNN was developed to automatically detect range shifts in simulated PGI data for head-and-neck cancer PBS treatment plans. The plans were generated based on data from 12 patients and one anthropomorphic head phantom; a standard PGI slit camera system was used to monitor one field per plan. A total of 386 scenarios representing various treatment deviations were simulated on planning and control CT scans. The scenarios were further assigned to different classes: non-relevant changes, relevant changes triggering treatment intervention due to range prediction errors, setup errors in beam direction, anatomical changes, or a combination [69]. Reliable PBS spots in conjunction with their Bragg peak data were utilized to create two 3D spatial maps containing PGI-determined range shift and proton number data. The team investigated 3 complexity levels of PGI data: optimal, realistic with added Poisson noise depending on the delivered proton number, and realistic with an additional positioning uncertainty of the camera. 3D-CNNs were trained at each complexity level using patient-wise leave-one-out cross-validation and evaluated on an independent test cohort. Results showed that CNNs were able to detect in-vivo range changes in realistically simulated PGI data. It was reported that the CNN ensemble achieved a binary accuracy of 0.95, 0.96, and 0.93 and a multi-class accuracy of 0.83, 0.81, and 0.76 for each complexity level, respectively [69]. Additionally, the model was able to accurately classify the nature of most of the sources of deviations.

A method to verify Bragg peak positioning in relation to the target without in-beam PET would be advantageous in clinical practice. Such a need stems from distribution modeling inaccuracies due to inconsistent considerations of Coloumbic interactions. A novel in-beam system was created to determine range by measuring scattered protons using scintillation detectors surrounding the body [70]. Similar to Pietsch *et al*, increasing simulation complexity levels were used to prevent model over-fitting [69]. To determine the MC dose, a simple simulation (A) of a rectangular phantom was irradiated. Subsequently, a DL model was trained to estimate 2D dose range from the values obtained through the first simulation. The DL models consisted of fully connected networks and CNNs. The following simulation (B) estimated 3D dose range, and included plastic scintillators around a rectangular phantom with an air layer. Lastly, a cylindrical phantom with internal structure closely mimicking a human body was simulated (C) and irradiated to confirm the previous estimation qualities. The resultant Bragg peak for simulation A was positioned within an error of 1.0 mm. In simulation B, the peak position was verified within 2.1mm. Finally, simulation C featured an estimated peak position error of 12.6 mm, which improved to 9.3 mm when the CT-derived mass density distribution was incorporated. In conclusion, both simulations A and B effectively approximated the 2D dose range within a 2 mm peak position error, which could potentially improve range estimation in clinical applications.

### 6.3. Dosimetry

Protoacoustic imaging specifically focuses on generating images based on the acoustic signals generated by protons. This application of protoacoustics is beneficial as it offers real time 3D dose verification, but the narrow angle of the ultrasound transducer is limiting. Jiang *et al*’s developed a DL model featuring a deep cascaded CNN (DC-CNN) to improve proton-acoustic image reconstruction using proton-acoustic signals detected by a matrix array [71]. The framework was validated on the IMPT plans of 81 patients with prostate cancer. A matrix ultrasound array was simulated near the patient treatment site to measure radiofrequency signals during dose delivery. To address the realism of the simulation, noise was added to the signals to consider tissue heterogeneity and attenuation. Trained on 204 samples and tested on 26, the DC-CNN-based model generated 3D pressures and dose maps. Results were qualitatively and quantitatively compared with the ground truth. The results showed that the proposed DC-CNN reconstructed high-quality 3D pressure images from the proton-acoustic images, potentially enabling 3D dose verification during treatment.

A deep learning based millisecond speed dose calculation algorithm (DoTA) capable of accurately predicting pencil beam proton dose depositions was created in 2022 [72]. The two current physics-based tools to calculate proton doses are analytical pencil beam algorithms (PBA) and MC simulations, which respectively feature better speed or precision. MC methods and PBAs are currently unable to synthesize the particle transport simulations in sub-second times necessary for next generation real-time adaptive radiotherapy. Accurate particle transport calculations are crucial as IMPT relies on MC simulations and PBAs to determine the spatial distribution of the physical dose delivered by the individual protons. Based upon previous long short-term memory (LSTM) networks, the proposed method sequentially calculates pencil beam dose distributions from relative SPR slices, and does not require a separate model per beam energy [72–74]. DoTA takes a novel approach by featuring an attention-based transformer backbone, which dispenses of convolutions and connects the encoder and decoder. This architecture is simpler than many other transduction models, which accounts for the additional speed that may benefit adaptive replanning.

With the parametric DoTA model, dose distributions from individual proton beamlets are able to be predicted from patient geometries (x) and beam energies (*ε*) with remarkable speed. The core concept underlying the model is that it extracts particle transport physics from the provided data and learns the function y(*θ*) = f(x, *ε*) through a series of artificial neural networks with parameters (*θ*). Performance was based on speed and accuracy comparison to standard clinically used methods, such as PBA or MC ground truth dose distributions. In summary, DoTA predicted dose distributions from single pencil beams 100 times quicker than widely used PBAs. The distributions yielded a gamma pass rate of 99.37 ± 1.17, close to standard MC accuracy in a fraction of the computational time. DoTA outperformed previous PBA and MC approaches with a 10% improvement in gamma pass rate, and features speed close to commercial GPU MC methods, critical for adaptive replanning events.

PBS proton therapy is particularly sensitive to heterogeneities, making successful dose prediction challenging. Zhang *et al* developed a DL-based workflow applicable to on-line adaptive proton therapy, supporting replanning if necessary [75]. The beam mask and sliding window methods are techniques designed to address significant tissue heterogeneities, and therefore improve the accuracy of DL models in predicting dosage. The beam mask is generated by raytracing the simulated proton beam paths through the body. The result allows the model to focus on regions directly impacted by the proton beams. The sliding window method processes smaller sections (windows) of the pCT data at a time, opposed to the entire volume at once. This method assists the DL model by concentrating on smaller, manageable areas of interest which slide across the entire volume, so the scan is analyzed in detail.

The proposed DL model included clinical PBS therapy plans from 186 patients, with 166 patients used for testing and 20 for training. Three experiments were conducted corresponding to three different methods. The first used conventional regions of interest (ROI), the second utilized the beam mask, and the third implemented the sliding window method. Afully connected 2D-Unet was adopted as the backbone, and the evaluation metrics included DVH indices, 3D Gamma passing rates, and dice coefficients to measure the correlation between the data sets. The predicted doses were compared to the ground truth doses. The calculation time for all three experiments was within 0.25s, demonstrating sufficient efficiency for online replanning and the potential to reduce initial planning time. Compared to the conventional experiment, both ML-guided trial methods improved the DVH indices. The conventional ROI method returned a DVH index of 0.74 ± 0.18 Gy, the beam mask method yielded 0.57 ± 0.21 Gy, and the sliding window method showcased the lowest value of 0.54 ± 0.15 Gy [RBE]. Similar trends were shown for the 3D Gamma passing rates: 96.93% ± 0.53% for conventional ROI, 98.88% ± 0.49% for beam mask, and 99.97% ± 0.07% for sliding window, along with the dice similarity coefficients. Implementing the sliding window method in a DL proton dose prediction framework for PBS therapy demonstrates promise in improved efficiency and accuracy compared to traditional ROI methods in adaptive scenarios.

As IMPT utilizes a narrow proton beam to target tumors and spare surrounding OARs, a margin of error is expected from patient setup and uncertainties in relative stopping power. During planning, additional margins are added to the treatment volume to mitigate these concerns, subsequently leading to higher doses to surrounding healthy tissue. Harms *et al* employed a deep-learning model to analyze daily CBCT images in order to derive relative stopping power, enabling online dose calculation [76]. Images collected from a DECT scanner from 23 head-and-neck cancer patients were utilized. The scans were converted to relative SPR maps then registered to the CBCT images taken on the first day. A CycleGAN-based architecture with a compound loss function was trained to synthesize a relative SPR map from a CBCT image. CT-based and CBCT-based SPR maps were compared using statistical analysis such as MAE and mean error, with the proposed method outperforming existing CT methods. The mean gamma passing rate was 94% when three-dimensional gamma index was calculated per plan, and 96% when gamma index was calculated per field [76]. The results of their study highlight the possibility for CBCT-guided adaptive dose planning for IMPT.

## 7. Future directions and limitations

Significant advances have been recently made incorporating ML into the proton therapy clinical workflow, but this is still an emerging area of investigation. While great strides have been made in methods applicable to all radiotherapy generally, proton therapy-specific models have been less frequently published. Areas of priority for future development are discussed in the following section.

### 7.1. Challenges

The recent adaptation of proton therapy compared to the history of data available for conventional photon therapy is a limiting factor in the incorporation of ML. There are many large, high-quality datasets available for photon therapy, but few deidentified and declassified proton therapy datasets. Additionally, for ML or AI to provide an adequate assessment of patient eligibility for varying treatment modalities, data from imaging, patient histories, dosimetric parameters, and treatment outcomes must be includedAchieving high generalizability of an ML model is challenging, especially when comprehensive patient data beyond institutional datasets is not readily available. Avoiding bias in model training is not unique to proton therapy, however there is also a lack of benchmark data from patients treated with proton therapy (i.e., proton therapy therapy dose distributions versus conventional dose distribution for dose prediction studies) compared to photon therapy.

ML models often act as a ‘black box’ without a transparent decision-making process. Clinical trustworthiness of ML-guided results is difficult to obtain without an interpretable reason behind treatment decisions. Particularly in the context of patient pre-screening and toxicity prediction, which inherently relies on multiple demographic, historical, qualitative, and quantitative patient factors, decision-making must evaluate a wide range of risks and potential outcomes. This ensures that all relevant factors are adequately considered in the decision-making process. Incorporating the humanness of such decision making is challenging to replicate with ML, although steps to create more clinically controllable and interpretable models are being taken [77].

Terunuma *et al* considered the possibility of using DL for real-time, patient-specific, markerless tumor segmentation in image-guided photon radiotherapy [77]. Their usage of attention-based augmentation emphasizes the significance of controllability and explainability in patient-specific models. ‘Controllability’ refers to how the clinicians are able to control where and how much attention the DL model places on different regions of the image, allowing for the prioritization of specific features or regions of interest during planning, such as soft-tissue over bony structures. On the other hand, ‘explainability’ refers to how and why the model makes the predictions it does, which improves interpretability and clinical trustworthiness. Additionally, a Gradient-weighted Class Activation Mapping technique was incorporated which generates a heatmap that illuminates the regions contributing most to the model’s predictions. The study found that combination of such techniques allowed for accurate tumor localization without the need for markers in a rapid manner. On 10 patient cases, the training time for the CNN was 30 min with a short inference time of 8 milliseconds. Regarding auto-segmentation of the thoracic cavity through inspiration and expiration phases, ‘the estimated three-dimensional 95 percentile tracking error, Jaccard index, and Hausdorff distance were 1.3–3.9 mm, 0.85–0.94, and 0.6–4.9 mm, respectively’ [77]. This study offers feasible ML advancements that could be applied to proton therapy, particularly in cases like head and neck cancer, where precise segmentation of soft tissue tumors from surrounding bony structures is essential for effective treatment. Such an application may be appropriate to both planning and adaptive segmentation for proton therapy.

Development of models for treatment planning are hindered by proton therapy data scarcity for model training. Vision models which address tasks such as segmentation and synthetic image creation rely heavily on expansive and accurately labeled datasets. Scans with varying patient anatomy and tumor types are necessary to train a model devoid of bias or overfitting. The computational demands of models frequently seen applied to dose or range calculation, such as GANs, require substantial resources many clinics may not be equipped with. Studies like that of Uh *et al*, which address complex imaging challenges such as bowel gas pockets, highlight the potential of ML in handling dynamic treatment conditions [56]. However, adapting ML models to account for such visual complications remains inherently challenging and may compromise the accuracy of predictions.

Models focused on adaptivity in proton therapy are currently the most prevalent application in the workflow. Adaptivity addresses real-time or inter-fractional patient anatomy and dose parameter changes. Image quality of CBCT scans, which are frequently used as a low-dose daily imaging strategy to ensure patient positioning and tumor size prior to treatment, suffer from poor quality. However, research on ML applications suffer from similar visual limitations when training and testing models. To create accurate reference 4D CBCT-pCT pairs for training, reliance on deformation image registration introduces dependency on its accuracy [61]. While ML methods have been utilized for in-vivo range verification to improve the SNR in prompt-gamma imaging, there are still challenges pertaining to optimizing the setup. For instance, optimizing the detector-to-phantom distance and reducing neutron-induced background remains one of the challenges for further enhancing prompt-gamma imaging for range verification [67].

The primary challenge in adapting existing photon therapy ML models to proton therapy arises from the innate difference in physics. Proton therapy harnesses the complex power of the Bragg peak to provide targeted therapy with decreased side effects. However, with such benefit comes difficulty in applying existing ML models. ML applications are common in other areas of radiation therapy, but the challenge in adapting and applying them to proton therapy is inherent to the predicate physics and hardware differences. While not specific to proton therapy and applicable to all forms of radiotherapy, an additional challenge that AI/ML innovations face in medical physics is designation as a software as a medical device (SaMD). The International Medical Device Regulators Forum (IMDRF) terms SaMD as ‘a software intended to be used for one of more medical purposes that perform these purposes without being part of a hardware medical device’ [78]. In the context of proton therapy, most models created to improve treatment outcome by improving efficiency and accuracy of varying stages of the clinical workflow fall into this definition. The challenges that continue to accompany this designation have been discussed by the IMDRF, who raise concerns such as uncontrolled distribution outside the control of the manufacturer [78]. Additionally, once commercialized, updates provided by manufacturers are often left in the hands of the medical device user to install. One of the primary challenges ML faces in commercialization and industry are adaptability and replicability; maintaining a consistent function across institutions with varying hardware applications is critical to providing consistent, quality care.

### 7.2. Quality assurance

At present, ML-assisted QA methods are sparse in proton therapy. QA is laborious in all high-precision radiation therapy modalities, and comprises management of beam delivery mechanisms, beam parameters, and instrumentation. Currently, ML models have been applied to improving gamma pass rates and predicting beam data in proton therapy, but no publications directly address ML for proton QA. Prerequisites for a quality plan, such as accurate delineation of the target and OARs, is beyond the scope of most QA procedures [25]. According to AAPM Task Group 224, QA procedures have been outlined for three proton therapy techniques: scattering, uniform scanning, and PBS [79]. This is an area of work where ML may be incorporated to improve accuracy, efficiency, and consistency in QA. Ono *et al* reported on the applications of AI for QA in radiotherapy as of 2024, and proton therapy is absent [80]. Current focus areas include feature extraction and selection, mechanical hardware setup QA, and gamma passing rate calculations.

### 7.3. Cost calculation

A major barrier to proton therapy treatment is cost. Proton therapy is expensive and is even more inaccessible to patients not geographically located near a treatment center. A National Cancer Database analysis reported that patients residing in a ZIP code with a median income of less than $46,000 per year had a statistically decreased chance of receiving proton therapy compared to those of higher income brackets [81]. By training on patient geographic and socioeconomic data and associated treatment outcomes, ML could be leveraged to improve equity or personalize the costs of proton treatment. Verma *et al* investigated the health economics of proton therapy in terms of sustainability and cost-effectiveness [82]. As this study was conducted purely from a health economics perspective, ML was not utilized. A significant concern brought to attention is the inherent inaccuracy of cost-effectiveness analyses due to the inability to account for all aspects of operation, such as electricity, equipment maintenance, treatment duration, and number of patients treated. The group reported that Markov and MC modeling have been utilized to address cost tabulations and comparisons, but all modeling studies have limitations due to probabilistic assumptions. As of 2016 when the study was conducted, the group reports that the routine treatment of breast cancer with proton therapy was not cost-effective. ML may greatly improve the accuracy and efficiency of cost-effectiveness analyses by reliably accounting for additional factors otherwise neglected through manual calculation [82]. Additionally, as demonstrated in previously discussed studies, ML is able to improve MC simulations in various aspects [47, 69, 72]. However, such an approach has not yet been accounted for in proton therapy economic analysis.

### 7.4. Outcome studies

Prognostic research is sparse in the context of both proton therapy and ML. The recent adaptation of proton therapy compared to the long history of conventional photon therapy is a primary limiting factor. ML has been utilized to predict toxicity and side effects [26, 27, 29], but not overall treatment prognostics in proton therapy. The integration of radiomics and circulating tumor cell counts has demonstrated improved accuracy in predicting the risk of reoccurrence for NSCLC patients treated with SBRT. Jiao *et al* accomplished this feat by building ML models on clinical measures and CT data from 421 NSCLC patients [83]. The models were able to not only predict recurrence risks but were able to stratify patients into specific outcome subgroups based on the CTC counts, achieving a concordance index of 0.88. Such a technique may be beneficial when considering adaptive replanning.

## 8. Conclusion

Many variations and applications of machine learning have appeared in the proton therapy workflow over the past five years with promise of improving efficiency, accuracy, and efficacy of proton therapy treatments. We have provided here a systematic overview of various ML methods and their role in proton therapy treatments and workflow. Several conclusions can be pulled from this review. U-Net architectures are prevalent in the patient pre-screening process, while convolutional neural networks (CNNs) and CycleGANs are more common in dose and range prediction applications for treatment planning. For adaptive monitoring, advanced =architectures such as general deep inception convolution neural networks (GDI-CNNs) and deep cascaded CNNs (DC-CNNs) have been used for real-time dose verification and range monitoring. However, certain areas such as cost analysis and quality assurance measures lack notable ML contributions and are areas of potential future interest. In conclusion, with the increased clinical interest in proton therapy, ML models have been increasingly developed for both clinical and research applications.

Table 1 Datasheet summary of current ML applications and associated data sources at various stages of proton therapy clinical treatment.

**Table 1.** Summary of recent proton therapy clinical applications of ML models.

Clinical application	References	ML model	Dataset	Model task
Pre-screening	Kouwenberg *et al* (2021)[22]	Gaussian naïve Bayes classifier	45 head and neck cancer patient photon plans	Pre-determine eligibility for IMPT and auto-plan if eligible
	Huet-Dastarac *et al* (2023)[23]	AI-PROTIPP (U-Net CNN)	60 oropharyngeal cancer patient proton and photon plans	Advise which radiation modality results in least adverse effects
	Chen *et al* (2024)[24]	Two 3D U-Nets	95 localized prostate cancer patients	Guide photon or proton plan partiality from NTCP models and dose prediction
	Geng *et al* (2023)[25]	KBP ML model	NSCLC patients	Compare effectiveness of IMPT versus IMRT plans
	Chamseddine *et al* (2023)[26]	Shallow CNN, Ensemble learning (CNNE)	117 hepatocellular cancer patient proton and photon plans	Liver toxicity prediction to guide modality selection
Toxicity Prediction	Padannayil *et al* (2023)[27]	Unsupervised k-means clustering	44 head and neck cancer patient dose surface histograms	Optimize proton beam placement to decrease skin irradiation based
Plan Optimization	Shafai-Erfani *et al* (2019)[41]	3D CycleGAN	50 base-of-skull tumor MRI and CT pairs	Generate sCT images from MRI
	Chen *et al* (2022)[42]	3D and 2D U-Net GANs	206 nasopharyngeal carcinoma patients MR and paired CT images	Generate sCT images from MRI for IMPT
	Zimmerman *et al* (2022)[43]	3D U-Net with ResNet-Blocks	47 meningioma patient MRI sequences and pCT data	Generate sCT images independent of MRI sequence data
	Borderias-Villarroel *et al* (2023)[44]	Four KBP ML models	60 oropharyngeal cancer patients	Optimize post-processing and dose-mimicking for ML-created IMPT plans
	Bruggen *et al* (2023)[45]	MLO	25 oropharyngeal cancer patients	Generate IMPT plans
Target Segmentation	Nielsen *et al* (2023)[35]	nnU-Net (CNN), deformable image registration, local AI	63 head and neck cancer patients	Contour OARs for proton plans
Dose Calculation	Wu *et al* (2021)[47]	3D HD U-Net	290 (90 head and neck, 93 liver, 75 prostate and 32 lung) cancer patients	Conversion of a PBS dose to a MC dose to boost accuracy of PB dose calculation
	Liu *et al* (2023)[48]	PSMC-Net (CNN)	Range of 33 clinical-level beam energies (75–225 MeV)	Optimize calibration of nominal energy and energy spread parameters in PSMC
	Grewal *et al* (2020)[50]	Gaussian process regression (GPR), shallow neural network (SNN)	4,231 Randomized patient QA measurements	Predict OF and MUs
	Stasica *et al* (2023)[52]	Deep CNN (VGG-16)	ImageNet database, 2,899,816 proton clusters, 853,717 electron clusters, 3,143,149 photon clusters	Accurately recognize protons and validate LET spectra
	Gao *et al* (2024)[53]	Supervised CycleGAN	50 prostate cancer patient dose maps	Predict LET distribution map for proton SBRT
	Tang *et al* (2024)[54]	3D Cascaded UNet	275 4-field prostate proton SBRT plans, rendering 1,100 fields	Calculate dose-averaged LET
Range Calculation	Ranjith *et al* (2024)[55]	Artificial neural network (ANN)	9000 proton spots	Predict beam spot size in varying axial positions
	Uh *et al* (2023)[56]	CycleGAN with deformable image registration	CBCT, same-day CT, and pCT of 81 pediatric patient abdominal cavities	Estimate proton ranges through abdominal cavity gas pockets by generating sCT images from CBCT
	Lee *et al* (2023)[57]	2D residual U-Net	8,659 image pairs from MC simulations	Predict beam range and spread-out Bragg peak
HU-to-SPR	Wang *et al* (2021)[30]	ccGAN	20 head and neck cancer patient DECT images	Predict SPR maps from DECT images
	Chang *et al* (2023)[31]	PDMI (Physics-constrained DL-based multimodal imaging), Supervised ResNet	7 tissue substitute MRI phantoms	Generate mass density maps from MRI and DECT
	Charyyev *et al* (2022)[33]	Residual attention GAN	70 simultaneous SECT/DECT images from head and neck cancer patients	Develop DECT images from SECT
	Wang *et al* (2022)[34]	ccGAN	195 pediatric brain tumor pCT and MRI	Create synthetic SPR maps from MRI sequences
Adaptivity	Wang *et al* (2022)[59]	CycleGAN with self-attentuation	125 pediatric brain tumor CT and T1-weighted MRI	Identify patients that would benefit from adaptive replanning due to anatomical variations
	Elmahdy *et al* (2019)[60]	CNN	20 prostate cancer patient manually-delineated images and deformation vector fields	Online recontouring of bladder and prostate from CT scans of IMPT patients
	Thummerer *et al* (2022)[61]	Deep CNN	45 thoracic cancer patient 4D-CBCTs	Convert sparse view 4D-CBCTs into 4D-sCTs
	Zhang *et al* (2022)[63]	SWFT-Net (DL)	1,706 head and neck cancer patient data	Increase speed of plan adjustment due to anatomical changes
	Wang *et al* (2023)[65]	Device-specific stack autoencoder (SAE) denoising models	512 raw proton pulses to phantoms	Denoise acoustic signals and reduce Bragg peak range uncertainty in-vivo
	Jiang *et al* (2023)[66]	General Deep Inception CNN (GDI-CNN)	1500 protoacoustic pulses	Denoise acoustic signals in-vivo
	Lerendegui-Marco *et al* (2022)[67]	Boosted decision trees, ANN	5 × 10^6^ simulated gamma-ray events between 200 keV and 7 MeV	Identify full-energy gamma events and enhance signal-to-total ratio in-vivo
	Pietsch *et al* (2023)[69]	3D-CNNs	12 head and neck cancer plans, 1 head phantom, 386 simulated treatment deviation scenarios	Automatically classify and detect range deviations
	Sato *et al* (2023)[70]	Fully connected DL models and CNNs	Total 10,083 simulated proton beams between 120–220 MeV	Verify Bragg peak positioning without in-beam PET
	Jiang *et al* (2022)[71]	Deep cascaded CNN	204 prostate cancer radiofrequency signals	3D dose verification *in vivo*
	Pastor-Serrano *et al* (2022)[72]	DoTA (DL based millisecond speed dose calculation model)	30 CT scans from prostate, lung and head and neck cancer patients	Predict pencil beam dose distributions in real time
	Zhang *et al* (2024)[75]	2D-Unet	186 PBS patient therapy plans	Predict dose from pCT data
	Harms *et al* (2020)[76]	CycleGAN	23 head and neck cancer DECT images	CBCT-guided adaptive IMPT dose planning

## Data Availability

No new data were created or analysed in this study.
